# *Pseudomonas* spp.-based synthetic microbial community effects on nutrient dynamics in citrus plants under induced dysbiosis

**DOI:** 10.1007/s00425-026-05205-6

**Published:** 2026-10-29

**Authors:** Toi Ketehouli, Mason R. Trub, Erica M. Goss, Samuel J. Martins

**Affiliations:** 1 Department of Plant Pathology, Institute of Food and Agricultural Sciences, University of Florida, Gainesville, FL 32611 USA; 2 Department of Microbiology and Cell Science, University of Florida, Gainesville, FL 32603 USA; 3 Emerging Pathogens Institute, University of Florida, Gainesville, FL 32610 USA

**Keywords:** Antibiotic, Microbiome, Phytobiome, Potassium, Sustainable agriculture, Synthetic bacterial community (SynCom)

## Abstract

**Main Conclusion:**

A targeted *Pseudomonas*-based synthetic microbial community reshaped rhizosphere bacterial communities and altered potassium partitioning under stress conditions, suggesting a potential role in mitigating antibiotic-induced dysbiosis.

**Abstract:**

Sustainable agriculture primarily relies on biological practices to improve crop yield and resilience. Synthetic bacterial communities (SynComs) are a promising approach to promote plant growth; however, their effectiveness in situ is influenced by interactions with native microbial ecosystems and by environmental stressors, such as low pH and antibiotic use. In this context, beneficial microorganisms like *Pseudomonas* may enhance nutrient availability and mitigate antibiotic-induced dysbiosis by supporting microbial community reassembly and functional stability, thereby improving plant health. This study evaluated the effects of a *Pseudomonas*-based SynCom on nutrient accumulation and rhizosphere microbial dynamics in citrus plants subjected to streptomycin-induced dysbiosis and low-pH conditions. Selected *Pseudomonas* strains, chosen for their compatibility and adaptability to low-pH conditions, and through whole-genome sequencing, were applied at equal concentrations (OD_600_ = 0.1). SynCom treatment increased Chloroflexota abundance in the rhizosphere while reducing Actinomycetota and Pseudomonadota after 24 days. By Day 45, the abundance of Actinomycetota decreased, with slight increases in the other two phyla. At the genus level, SynCom application reduced the abundance of *Pseudomonas* and *Agrobacterium*, while enriching the *Klebsiella* community. Plants treated with SynCom under stress exhibited a 25% increase in leaf potassium levels, accompanied by a 29% decrease in root potassium (essential for stomatal regulation, osmotic adjustment, and photosynthetic efficiency, which are fundamental to tree growth and stress resilience) levels. Overall, our findings demonstrate that a targeted *Pseudomonas*-based SynCom can reshape rhizosphere communities and improve nutrient dynamics under abiotic stress, highlighting its potential as a sustainable strategy to mitigate antibiotic-induced dysbiosis and enhance citrus resilience.

**Supplementary Information:**

The online version contains supplementary material available at https://doi.org/10.1007/s00425-026-05205-6.

## Introduction

Modern agricultural systems are facing increasing problems linked to microbial dysbiosis, an imbalance in plant-associated microbial communities, often shifting communities toward increased stochasticity and reduced functional stability (Thomas et al. 2026). Excessive fertilizer application, pesticide use, soil acidity, and the employment of broad-spectrum antibiotics can undermine beneficial microbial networks crucial for nutrient cycling, disease suppression, and plant resilience (Timmis and Ramos 2021).

In response to these challenges, researchers have increasingly focused on designing and applying synthetic microbial communities (SynComs) composed of beneficial microorganisms with plant growth-promoting traits (PGPTs) (Shayanthan et al. 2022; Martins et al. 2023; van Leeuwen et al. 2023). SynComs are designed assemblages of multiple microbial species that provide targeted benefits to plants, including promoting growth, increasing nutrient update, and promoting stress resilience (Martins et al. 2023; van Leeuwen et al. 2023). Microbiome engineering is an approach to modulate the rhizosphere to desired functions under challenging environments (Herrera Paredes et al. 2018). The theoretical basis for SynComs assumes that plant health arises from interactions between the plant and its associated microbiome, particularly those residing in the rhizosphere (Hacquard et al. 2015). The rhizosphere (soil region influenced by root exudates) is an essential hub for microbial activity and interactions, which play crucial roles in nutrient cycling, pathogen suppression, and plant hormone regulation (Philippot et al. 2013).

In the context of examining microbiome stability under agricultural stressors, citrus systems can serve as an effective model for analyzing how significant disturbances alter plant–microbe interactions. Streptomycin, an antibiotic historically registered for agricultural use since the early 2000 s, was applied in Florida citrus under emergency exemptions beginning in 2016, and its use was expanded through an EPA registration amendment in January 2021 to manage bacterial diseases, including huanglongbing and citrus canker till its ban in February 2024 (Archer et al. 2020; Batuman et al. 2024). Regardless of application context, its broad-spectrum activity can inadvertently disrupt beneficial microbial communities, particularly those involving *Pseudomonas* and *Streptomyces* spp., which play essential roles in nutrient cycling, disease suppression, and plant stress tolerance (Zhang et al. 2013; Liu et al. 2020; Ketehouli et al. 2024). Moreover, acidic (low pH) soil conditions, common in several citrus-growing areas, exacerbate plant growth and microbial stability by hindering nutrient availability and microbial function (Morgan and Graham 2019; Li et al. 2022). Soil acidification has emerged as a growing threat to sustainable citrus production, especially in subtropical regions where long-term fertilizer use and acid rain lower soil pH and impair nutrient availability (Guo et al. 2010; Li et al. 2022). Acidic pH conditions inhibit root development and microbial activity, increase heavy metal toxicity, and modify rhizosphere microbial composition, thus further compromising plant health and production (Rousk et al. 2010). The combination of low pH and antibiotic exposure could generate a complex environmental stress that poses concerns to both plants and their associated microbial communities.

Among the diverse array of beneficial microorganisms found in the rhizosphere are plant growth-promoting rhizobacteria (PGPR) (Bhattacharyya and Jha 2012). *Pseudomonas* spp. are widely studied for their ability to produce phytohormones, solubilize phosphate, suppress pathogens through secondary metabolites, and improve plant resilience to abiotic and biotic stressors (Santoyo et al. 2021; Choudhary et al. 2022). *Pseudomonas* spp. possess various traits that can directly or indirectly support plant health, including the production of antibiotics, siderophores, and phytohormones (Singh et al. 2022; Mehmood et al. 2023). Some *Pseudomonas* spp. are also known for their ability to induce systemic resistance in plants, a phenomenon in which resistance to a wide range of pathogens is enhanced following localized exposure to a beneficial microbe (Pieterse et al. 2014).

Understanding the interactions between SynComs and citrus plants, as well as the native microbiome under cumulative stress, is essential for developing resilient, sustainable microbiome-based solutions. Here, we hypothesized that a *Pseudomonas*-based synthetic microbial community (SynCom) would restore functional rhizosphere microbiome structure and enhance nutrient homeostasis under low soil pH and in the presence of antibiotics, which induce dysbiosis. The objective of this study was to assess the impact of *Pseudomonas* SynComs on the rhizosphere microbiome and nutrient absorption of citrus plants under dysbiosis.

## Materials and methods

### Plant materials, soil sample collection, and experimental design

A greenhouse experiment was established at the University of Florida in Gainesville, Florida, USA (54 m altitude, 29°63′90''N29°63''N and 82°35′60''W82°35′60''W) at 28 °C and 60–70% humidity. Six-month-old citrus seedlings (US-942) that were purchased from Agromillora, Florida, were cultivated in 2 L pots that were filled with HFC/B growing mix (bought from HydraFiber Advanced Substrate, Buffalo Grove, IL, USA) for four months. The plants were then transplanted to new 2-L pots containing citrus grove soil collected from the Plant Science Research and Education Unit—UF IFAS PSREU Citra, FL; 29.4089563N − 82.1708277W, and cultivated for 6 months. The results of soil physicochemical analysis, which includes the organic matter content, macro- and micronutrients are as follows: Cu^2+^(2.94 mg kg^−1^), Mn^2+^(32.58 mg kg^−1^), Ca^2+^(2722.07 mg kg^−1^), Zn^2+^(24.65 mg kg^−1^), Mg^2+^(422.54 mg kg^−1^), K^+^(180.49 mg kg^−1^), P (569.79 mg kg^−1^), NH_4_^+^(3.99 mg kg^−1^), NO_3_^−^(3.71 mg kg^−1^), 5.38% of organic matter, and a pH equal to 6.79. All the plants were watered every three days before and during the treatments. The plants were placed in a growth chamber at 25 °C, 70% humidity, and a 16-h photoperiod, with 250 µM m^−2^ s^−1^ provided before treatments.

A randomized complete block design (RCBD) with five biological replicates per treatment was used. Six treatment groups were established: Control (plants that received water), Str (plants treated with streptomycin), SynCom (plants inoculated with a suspension of four *Pseudomonas* isolates as SynCom), All treatments (Str + pH + SynCom: plants treated with streptomycin, subjected to low pH, and inoculated with the SynCom), Str + pH (plants administered streptomycin and exposed to low-pH conditions), and Str + SynCom (plants subjected to streptomycin treatment prior to SynCom application).

To induce acidic stress conditions, the pH solution (Hoagland nutrient with adjusted pH = 3.5 using 1 M of HCl) was applied on experiments Day 1 and 4 to All treatments and Str + pH groups, and the remaining groups were given the same volume of Hoagland at pH = 7 (200 mL) (see Table S1 for experimental schedule of applications and sampling during the 45-day study). The Hoagland nutrient solution was composed of 2.5 mM Ca(NO_3_)_2_, 2.5 mM KNO_3_, 1 mM MgSO_4_, 0.5 mM KH_2_PO_4_, 20 μM Fe-EDTA, 10 μM H_3_BO_3_, 2 μM MnCl_2_, 2 μM ZnSO_4_. This pH level was chosen to exert robust, regulated abiotic stress, intended to markedly disrupt plant physiology and rhizosphere microbial populations. This level was designed to simulate significant edaphic stress alongside antibiotic-induced dysbiosis, rather than to reflect typical field conditions. From Day 7, for relevant groups, treatments were administered at 24-h intervals in the following order: we applied 150 mL of pH solution (All treatments and Str + pH groups), followed 24 h later by 150 mL of Str treatment (600 mg of streptomycin was dissolved in 1 L of sterile filtered tap water, applied to Str, All treatments, Str + pH, and Str + SynCom groups), and 100 mL of SynCom solution 24 h after the streptomycin application (SynCom, All treatments, and Str + SynCom groups). This sequence was repeated every 7 days for 45 days, for a total of 6 successive treatments. Plant roots and rhizosphere soil were collected at 7, 24, and 45 days after the first pH stress induction.

### Pseudomonas species isolation

We collected rhizosphere samples from organic citrus groves in Clermont, Florida, to isolate *Pseudomonas* spp. from rhizosphere soil. Twenty-one rhizosphere samples were obtained from three different citrus varieties (Sugar Belle on US 897 rootstock, *n* = 7, Valencia and Navels on Flying Dragon rootstock, *n* = 14). For each tree, fine roots located roughly 20–30 cm from the trunk and at a soil depth of 10–20 cm were meticulously dug using a sterilized hand trowel. Any loosely adhering bulk soil was carefully removed, and soil firmly attached to the root surface was brushed into sterile 50 mL tubes to represent the rhizosphere portion. The samples were then transported on ice and processed the same day. We weighed 1 g of each rhizosphere soil sample in the laboratory and suspended it separately in 9 mL of phosphate-buffered saline (PBS) to create a 1:10 dilution. The mixtures were vortexed thoroughly to ensure proper suspension of the rhizosphere soil particles. We performed serial dilutions by transferring 1 mL of the initial soil suspension into Eppendorf tubes containing 9 mL of PBS, continuing this process to create a series of dilutions typically ranging from 10^–1^ to 10^–6^. We plated 100 µL from the middle dilutions (10^–3^ and 10^–4^) onto sterile Petri dishes containing King’s B medium supplemented with novobiocin (45 μg mL^−1^), penicillin (75 units mL^−1^), and cycloheximide (75 μg mL^−1^). We incubated the plates at 28 °C for 24–48 h. We selected the colonies that exhibited typical *Pseudomonas* characteristics (small, round colonies that produce a fluorescent pigment (pyoverdine) under UV light on King’s B medium) and streaked them onto fresh plates to obtain pure cultures. We subsequently stored the pure cultures of 30 isolates in 20% glycerol at − 80 °C fridge.

### pH test for *Pseudomonas* isolates

The pH tolerance of bacterial isolates was assessed following a modified version of the protocol described by Aranda-Díaz et al. (2020) and Schubiger et al. (2020). The bacterial isolates were removed from − 80 °C fridge and placed on ice. 30 μL of each isolate was used for growth in King B liquid medium (initial pH approximately pH 7 ± 2) at 28 °C with shaking for 24 h. The cells were harvested by centrifugation at 8000 g for 5 min. The supernatants were discarded, and the pellet from each isolate was dissolved in 0.01 M PBS. We thereafter measured the optical density (OD_600_) of each solution to standardize the bacterial inoculum. We adjusted the optical density (OD_600_) to 0.6 for all the isolates. For the pH assay, 200 μL of each King B medium adjusted to different pH values (3.5, 5.5, 6, 7.5, 8, 8.5) was transferred into individual wells of a 96-well microplate. We transferred 30 μL of standardized bacterial suspension into each well, incubated at 28 °C in a shaker at 80 rpm for 24 h, and monitored bacterial growth by measuring absorbance at OD_595_ wavelength using a microplate reader as described by Schubiger et al. (2020). All assays were performed in triplicate. The growth patterns of selected *Pseudomonas* isolates under different pH conditions are shown in Fig. S1.

### Bacterial compatibility and pre-identification analyses

We performed BOX-PCR (Repetitive Extragenic Palindromic PCR) to assess the isolates’ genetic diversity and to generate genotypic fingerprints. Genomic DNA was extracted from each isolate using a standard bacterial DNA extraction kit [Quick-DNA Fecal/Soil Microbe Miniprep Kit from Zymo Research (Irvine, CA, USA)] according to the manufacturer’s instructions. PCR amplification using the primer BOXA1R (5′-CTACGGCAAGGCGACGCTGACG-3′) targets explicitly repeated BOX elements distributed across bacterial genomes (JLW 1998). Each 25 μL reaction comprised 1× PCR buffer, 2.5 mM MgCl_2_, 200 μM of each dNTP, 0.4 μM BOXA1R primer, 1 U Taq DNA polymerase, and approximately 50 ng of template DNA. Amplifications were performed in a thermal cycler using the following protocol: initial denaturation at 95 °C for 7 min; 35 cycles consisting of denaturation at 94 °C for 1 min, annealing at 52 °C for 1 min, and extension at 65 °C for 8 min; concluded with a final extension at 65 °C for 16 min. PCR products were separated by electrophoresis on 1.5% agarose gels at 100 V for 3 h in 1× TBE buffer, then stained with EZ Vision. Gel pictures were obtained under UV light, and banding patterns were evaluated manually as present (1) or absent (0). The resultant binary matrix was employed to assess genotypic diversity among isolates.

A cross-streak assay was conducted on King’s B agar plates to evaluate the compatibility of the 12 selected bacterial isolates (after BOX-PCR analysis). A single isolate (20 µL) was vertically streaked in the center of the plate to function as the reference strain. Eleven remaining isolates (4 µL each) were applied as distinct dots, oriented perpendicularly along both sides of the center stripe to prevent direct contact during inoculation. Plates were incubated at 28 °C for 48 h, following which growth interactions were assessed. Isolates that exhibited normal growth near the central streak were deemed compatible, but inhibition was noted when a distinct clear zone appeared between the streak and the spotted isolate (Table S2). This assay identified isolates suitable for co-cultivation in subsequent synthetic community (SynCom) construction. We extracted the DNA of each compatible isolate using a Quick-DNA Fecal/Soil Microbe Miniprep Kit from Zymo Research. We stored the DNA at − 20 °C until ready to proceed with submission to Azenta US, Inc. (Research Triangle Park, NC, USA) for Sanger sequencing of the V1−V9 regions of the 16S rRNA gene amplification using the specific primers 27 F and 1492R. The nucleotide sequence similarity of the 16S rDNA from these bacterial isolates was determined using the Blast tool at the National Center for Biotechnology Information (NCBI) and the GenBank database.

### Whole-genome sequencing and genome assembly of *Pseudomonas* isolates

Whole genomes of the four *Pseudomonas* bacterial isolates used for SynCom construction were sequenced using an Illumina NovaSeq X Plus sequencer in one or more multiplexed shared-flow-cell runs, producing 2×151 bp paired-end reads. Demultiplexing, quality control, and adapter trimming were performed with bcl-convert (v4.2.4), available from https://www.illumina.com. Raw reads are assembled using the methodologies outlined in Timilsina et al. (2019). Adapters were removed using Trim Galore with the standard parameters to produce clean sequences and pair raw reads (Martin 2011). Paired reads were assembled via SPAdes (v.3.10.1) with the 'cautious' option, and contigs less than 500 bp and with k-mer coverage below 2.0 were discarded. Verified reads were aligned to the filtered contigs utilizing the default parameters of Bowtie 2 (v. 2.3.3) (Langmead and Salzberg 2012). SAM files were transformed to BAM files via SAMtools (Li et al. 2009). Pilon refined the draft genome assemblies using the '–frags' configuration (Walker et al. 2014). Genome statistics were computed utilizing a Python script (https://github.com/sujan8765/nepgorkhey_python/blob/master/genome_stats.py). Pyani (v.0.2.10) was employed to calculate the average nucleotide identity (ANI) among assemblies. Digital DNA–DNA hybridization (dDDH d4) values were obtained using the Genome-to-Genome Distance Calculator (GGDC) web server (DSMZ; Meier-Kolthoff et al. 2022). ANI and dDDH were also calculated based on comparisons with reference strains available in public databases. Whole genome sequences and raw read data were deposited in the NCBI GenBank database under BioProject number PRJNA1280539.

### Secondary metabolite biosynthetic gene cluster (BGCs) prediction

The whole-genome sequence data of the four *Pseudomonas* strains were subjected to a prediction of secondary metabolite BGCs using the antiSMASH 8.0 bacterial version (accessed on May 14th, 2025, at https://antismash.secondarymetabolites.org) with the default settings of KnownBlusterBlast, ClusterBlast, SubClusterBlast, MIBiG (Minimum Information about a Biosynthetic Gene) cluster comparison, active siteFinder, RREFinder (RiPP Recognition Element), and Cluster Pfam analysis (Blin et al. 2025). The antiSMASH tool was designed to predict and annotate existing, novel, and undiscovered BGCs.

### Synthetic community (SynCom) assembly

To subculture the four bacterial strains, 20 μL of each isolate was streaked onto King’s B agar and incubated for 24 h. A single colony for each isolate was then inoculated in 25 mL King’s B liquid media, and shaken at 180 rpm and 28 °C for 48 h. After centrifuging and resuspending the pellet with PBS, the cultures were mixed to construct the SynCom. The concentration of each strain applied was estimated at OD_600_ = 0.1, and the strain ratio was SMN1:SMN5:SMV3:SMK4 = 1:1:1:1. The SynCom was applied to the soil in the corresponding treatments by drenching (see Table S1 for application details).

### Rhizosphere sampling and DNA extraction for microbial community analysis

At 7, 24, and 45 days after the first pH stress induction and other treatments, rhizosphere soil was collected from five distinct plants per treatment to encompass the complete diversity and minimize sample variability. Before sampling, we prepared an epiphyte removal buffer (30 mL per sample) by dissolving 6.75 g of KH_2_PO_4_, 8.75 g of K_2_HPO_4_, and 1 mL of Triton X-100 in 1 L of sterile water. The buffer was sterilized using a vacuum filter with a 0.22 μm pore size. We used sterile scissors to excise a representative segment of roots (fine, actively growing roots from the mid-root zone, where we think they exhibit strong rhizosphere activity and consistently harbor a representative microbiome) and transferred at least 500 mg of root tissue into a 50-mL conical tube. 30 mL of epiphyte removal buffer was added to immerse the roots, and the sample was promptly placed on dry ice, as specified by Simmons et al. (2018). We minimized root breaks that could lead to the leaching of endophytic bacteria into the rhizosphere. After that, the materials were stored at − 80 °C. We thawed the root sample on ice to separate the rhizosphere from the roots, then vortexed and sonicated the samples at 4 °C for 10 min. The roots were removed with sterile forceps. The tube containing buffer and rhizosphere was centrifuged for 10 min at 4 °C and 4000 g. The supernatant was decanted, and the tube containing the rhizosphere fraction was flash-frozen in liquid nitrogen and stored at − 80 °C until DNA extraction commenced. For each sample, 250 mg of rhizosphere soil was utilized for DNA extraction employing a Quick-DNA Fecal/Soil Microbe Miniprep Kit from Zymo Research. We preserved the DNA at − 20 °C until it was prepared for submission to SEQCENTER (Pittsburgh, PA, USA) for amplification and sequencing of the V3−V4 region of the 16S rRNA gene, utilizing the following primer sequences: 341F: CCTACGGGDGGCWGCAG; 806R: GACTACHVGGGTATCTAATCC (Klindworth et al. 2013). After clean-up and normalization, samples were sequenced on a P1 600cyc NextSeq 2000 Flow cell to produce 2×301 bp paired-end reads. Raw sequencing data were processed using bcl-convert (Illumina) for quality filtering and adapter trimming. Sequences were imported into Qiime2 for further analysis. Primer sequences were removed using the cutadapt3 plugin. Sequence denoising and quality control were performed using the DADA2 plugin in QIIME2 to generate high-quality sequences. Sequencing depth was generally comparable across samples, although some variation in library size was observed (see Fig. S6). For downstream taxonomic and statistical analyses, sequences were clustered into operational taxonomic units (OTUs) using the VSEARCH5 algorithm implemented in the feature-classifier plugin of Qiime2, based on the Silva 138 reference database (99% similarity threshold) (Callahan et al. 2016). OTU tables were further processed using the SHAMAN Pasteur Institute pipeline for normalization and statistical analysis (Volant et al. 2020), and their counts were adjusted to reflect their relative abundances within each sample. Raw read data are available at NCBI SRA under accession PRJNA1280539.

### Roots and leaves macro- and micro-nutrient content measurements

To assess how the SynCom influences plant nutrient uptake, we analyzed nutrient content in roots and leaves after the last treatments. Roots were collected by excising tiny segments from three distinct places within the root system (upper, middle, and lower areas) and amalgamated into a composite sample weighing roughly 2 g fresh weight. We collected four young, fully grown leaves from each plant, totaling approximately 1–2 g of fresh weight. The concentration of nutrients in plant tissue samples was determined using an inductively coupled plasma optical emission spectroscopy (ICP-OES) instrument, following standard plant tissue digestion and elemental analysis procedures (Jones Jr 2001) from the University of Florida, Analytical Services Laboratories (ANSERV Labs). Prior to analysis, 0.2 g of each dried and ground sample was weighed into digestion tubes. Two mL of concentrated nitric acid was added to each tube, and the samples were allowed to digest overnight. The samples were then heated to 125 °C for 2 h, after which an additional 2.5 mL of concentrated nitric acid was added. Following the cooling period, the samples were diluted to a final volume of 20 mL with deionized water. The prepared samples were subsequently analyzed using the ICP-OES instrument.

### Statistical analyses

SHAMAN, an online bioinformatics tool for processing raw reads in targeted metagenomics (https://shaman.pasteur.fr/), was used to assess bacterial 16S rRNA gene sequences using statistical methods. We generated bar plots of the microbial community and Shannon curves to characterize rhizosphere diversity. Principal component analysis (PCA) was conducted on normalized relative abundance data to elucidate trends in microbial community composition. The Bray–Curtis dissimilarity metric was used to measure variation in community composition between samples. Shannon Index values (Fig. S2) were computed to assess alpha diversity across treatments. Statistical differences in community composition were evaluated using permutational multivariate analysis of variance (PERMANOVA) based on Bray–Curtis distances and Wilcoxon tests for pairwise comparisons of diversity indices (*P* < 0.05) (Volant et al. 2020). Differential abundance at the phylum and genus levels was assessed using both model-based and nonparametric statistical approaches. For model-based analysis, raw phylum- and genus-level operational taxonomic units (OUT) count data were analyzed using the DESeq2 package in RStudio version 4.2.3. For each phylum and genus, treatment effects were evaluated using likelihood-ratio tests to assess overall differences among treatments. When significant overall effects were detected, pairwise comparisons between treatments were subsequently performed within the DESeq2 framework using Wald tests with specified contrasts. P-values were adjusted using the Benjamini–Hochberg method.

To investigate the impact of pH on each bacterial isolate’s growth, optical density (OD_595_) was measured at six pH levels (3.5, 5.5, 6.0, 7.5, 8.0, and 8.5), with three replicates per treatment. A one-way analysis of variance (ANOVA) was conducted for each isolate across pH levels to determine whether pH significantly influenced growth. When ANOVA revealed significant differences (*P* < 0.05), Tukey’s honestly significant difference (HSD) test was employed for pairwise comparisons among pH levels. The mean optical density and standard error were computed for each isolate at each pH level. Results were illustrated using bar plots created in R (version 4.2.3) with the ggplot2 tool (Fig. S1). Tukey group letters were presented directly on the graphs to emphasize statistically significant differences in isolate growth responses to pH levels.

## Results

### Genomic relatedness and taxonomic delineation of bacterial isolates

To evaluate the genomic relatedness and taxonomic classification of the four bacterial isolates, phylogenetic analyses, average nucleotide identity (ANI), and digital DNA–DNA hybridization (dDDH d4) were performed. Phylogenetic analysis using the GGDC web server (http://ggdc.dsmz.de/) and the DSMZ phylogenomics pipeline based on single-gene alignments positioned isolates SMN1, SMN5, SMK4, and SMV3 in proximity to *P. vlassakiae* RW4S2, *P. zarinae* SWRI108, *P. izuensis* IzPS43 3003, and *P. fulva* DSM 17717, respectively (Table 1). However, their ANIb and dDDH d4 values fell well below species-level thresholds, indicating that the four isolates do not correspond to any currently recognized species.

**Table 1:** Genome statistics, including genome size, N50 value, and GC content for four *Pseudomonas* strains used in SynCom

Identifier	Proximity species	Genome size (bp)	Number of CDSs	dDDH d4 (%)	GC content (%)	N50 (bp)	Number of contigs	Accession number
SMK4	*P. izuensis*	6,929,526	6,219	36.8	60.05	54,856	244	JBPKAM000000000
SMN1	*P. vlassakiae*	6,289,025	5,612	45.3	62.70	77,132	151	JBPKAL000000000
SMN5	*P. zarinae*	6,039,975	5,291	33.9	60.19	219,540	59	JBKGGX000000000
SMV3	*P. fulva*	4,761,226	4,258	54.8	60.42	136,775	58	JBPKAN000000000

**Fig. 1:**
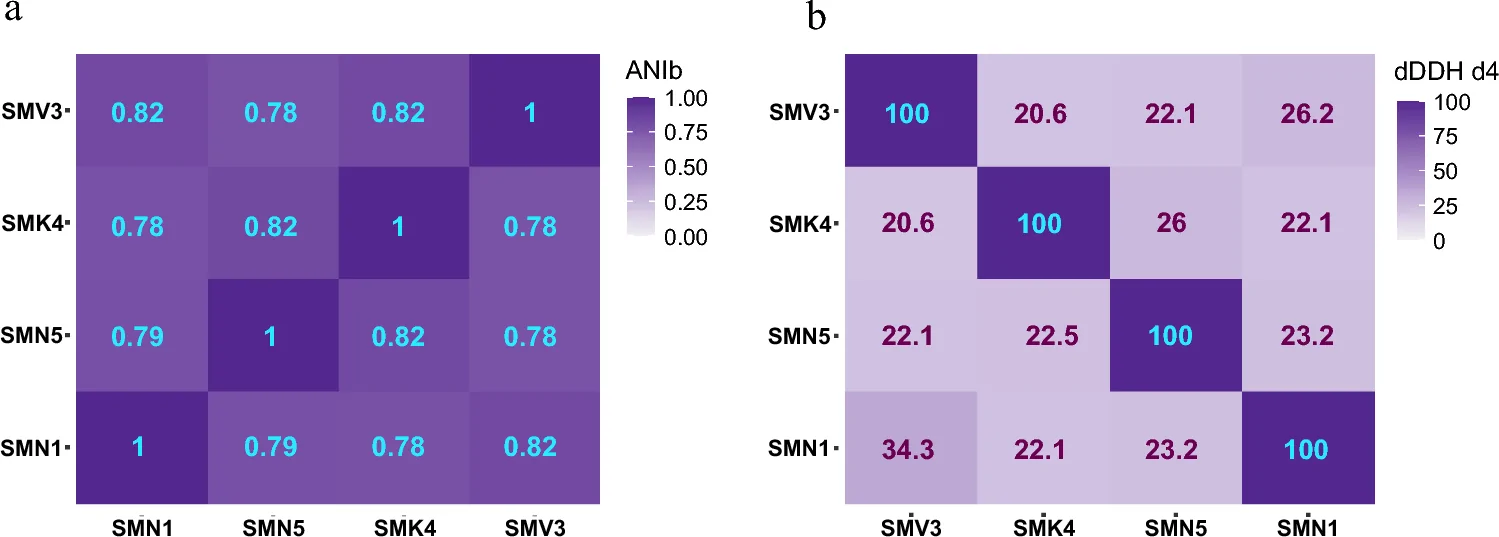
Comparative genomic analysis among four bacterial isolates. **A** The average nucleotide identity (ANIb) BLASTn-based heatmap shows pairwise genomic similarity; darker shades indicate greater similarity. **B** Digital DNA–DNA Hybridization (dDDH, formula d4) heatmap representing genomic relatedness; values ≥ 70% suggest species-level similarity. ANIb was calculated using the pyani package v.0.2.12 (Pritchard et al. 2016) on the HiPerGator high-performance computing system at the University of Florida, with alignment coverage shown in Fig. S5. The dDDH, d4 data were generated using the Genome-to-Genome Distance Calculator (GGDC) and the DSMZ phylogenomics pipeline provided by the Leibniz Institute DSMZ-German Collection of Microorganisms and Cell Cultures (https://ggdc.dsmz.de/) (Meier-Kolthoff et al. 2022). The heatmaps are made using RStudio version 4.2.3

Further confirming that the four strains represented distinct species, pairwise ANI values were below 95% (Fig. 1A). Similarly, the dDDH results (formula d4; Fig. 1B) showed isolate pairs with dDDH d4 values below the 70% threshold considered to belong to different genomic species, as shown in Fig. 1 and Table 1. Together, these analyses suggest the SynCom comprised four distinct *Pseudomonas* spp.

### Strain-specific and conserved biosynthetic gene clusters identified by antiSMASH

To explore the biosynthetic potential of the four *Pseudomonas* strains, BGCs were predicted and compared using antiSMASH 8.0 (Table 2). This analysis aimed to identify genetic potential to produce secondary metabolites (like siderophores and antimicrobial compounds) that may contribute to plant–microbe interactions and rhizosphere processes under stress conditions. A conserved hydrogen cyanide (HCN) gene cluster was detected in all strains (Region 1.1), indicating a shared potential for cyanogenesis. Additionally, strain SMN5 exhibited a second, unique HCN cluster (Region 6.1), suggesting possible strain-specific amplification of this trait. Non-ribosomal peptide synthetase (NRPS) clusters displayed considerable variation across strains. A viscosin-like cluster was detected with low similarity in SMN1 and SMN5, while Stechlisins B2-F-type NRPS clusters were strongly conserved in SMV3 and SMK4 (Region 4.1), indicating functional differentiation in secondary metabolite production. A ranthipeptide BGC (Region 14.2/1.4) was exclusive to SMN1 and SMN5, while a unique Ririwpeptide ABC cluster (Region 115.1) was found only in SMN1, pointing to strain-specific metabolite capabilities. Terpene-related carotenoid clusters (Region 38.1) and arylpolyene BGCs (Region 5.2) were identified exclusively in SMV3 and SMK4, suggesting shared metabolic traits between these two isolates. Additionally, an uncharacterized NRPS cluster (Region 13.1/34.1) was found in SMN5, SMV3, and SMK4, further supporting potential functional diversification. These findings highlight conserved and divergent BGC profiles among the strains, underscoring potential differences in metabolite production that could influence ecological interactions or plant-associated functions.

**Table 2:** Comparison of biosynthetic gene cluster (BGCs) types and similarities in the four bacterial strains using antiSMASH 8.0, revealing conserved clusters, strain-specific or subgroup-specific distributions, and similarity confidence levels based on antiSMASH cluster alignments. Similarity levels refer to the degree of match with known clusters in the MIBiG database: High, suggest strong gene synteny and high sequence identity; likely to produce the same or closely related secondary metabolite; Medium represents partial gene similarity or disrupted synteny; may create a structural variant or analog of a known compound; and Low denotes a weak homology; product that may be novel or divergent from known metabolites. Region indicates biosynthetic gene cluster identifiers, with entries X.Y indicating cluster numbers (Y) on contig (X), aiding in tracking conserved or unique BGCs during comparative genomics

		(BGC and similarity)				
Region	Gene type	SMN1	SMN5	SMV3	SMK4	Notes/observations
1.1	Hydrogen cyanide	High	High	High	High	Conserved across all strains
4.1	NRPS	Viscosin (Low)	Thanafactin A (Low)	Stechlisins B2-F (High)	Stechlisins B2-F (High)	Same cluster name in strains SMV3 and SMK4
14.2/1.4	Ranthipeptide	Present	Present	–	–	Only in strains SMN1 and SMN5
25.1/46.1	NRPS (Pyoverdine SXM-1)	Low	Medium	–	–	Variable similarity confidence
115.1	Ririwpeptides ABC	High	–	–	–	Unique to strain SMN1
38.1	Terpene (carotenoid)	–	–	High	High	Only present in strains SMV3 and SMK4
5.2	Arylpolyene	–	–	APR vf (Low)	APR vf (Low)	Specific to strains SMV3 and SMK4
13.1/34.1	NRPS (no match)	–	Present	Present	Present	Uncharacterized NRPS clusters
6.1	Hydrogen cyanide	–	High	–	–	Only strain SMN5 has an additional HCN cluster

### Effect of SynCom and pH on the bacterial communities in the rhizosphere of plants under dysbiosis

**Fig. 2:**
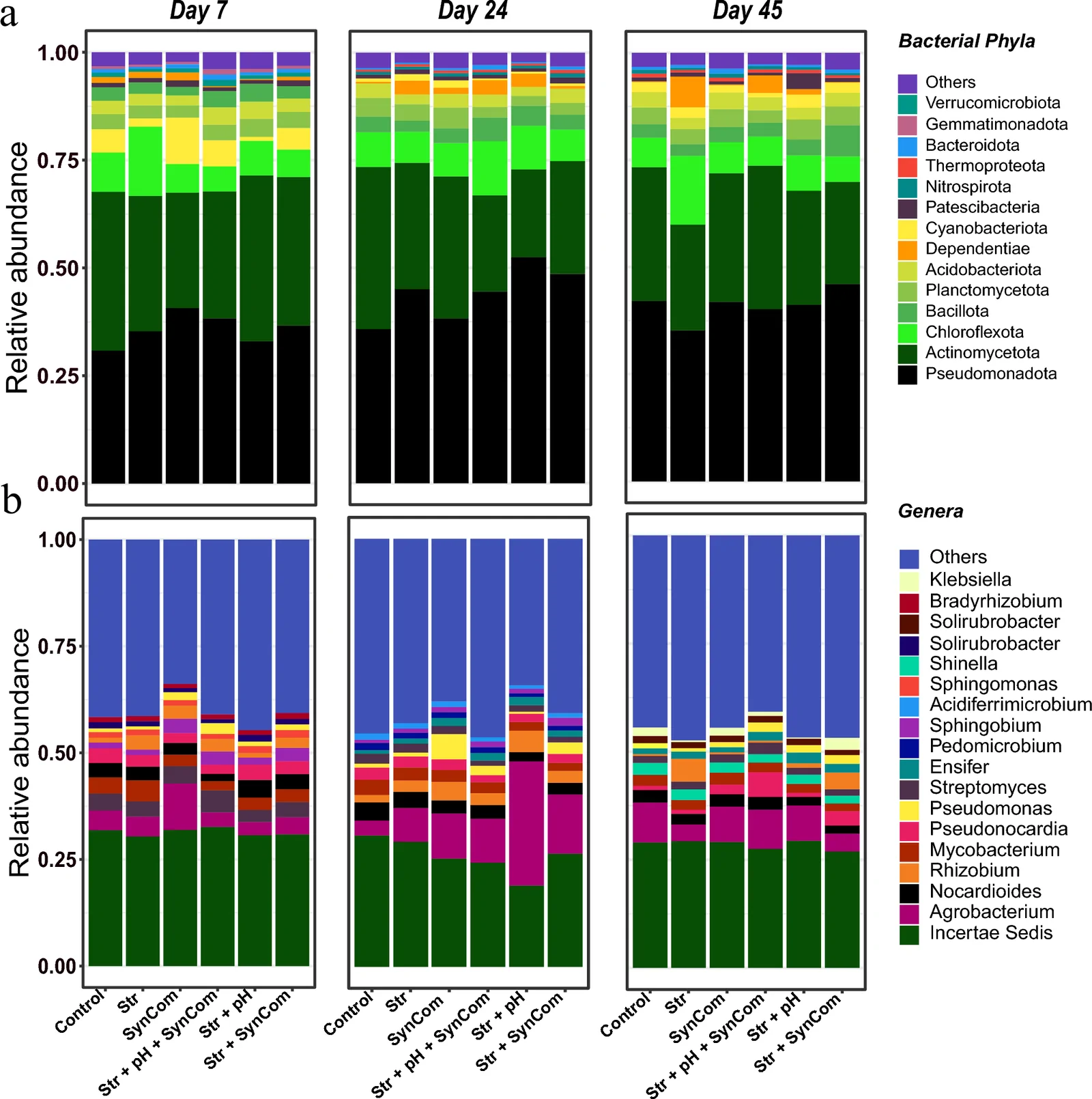
Relative abundance of dominant bacterial taxa under different treatments over time.** A** Barplot of dominant bacterial phyla. **B** Genera in the rhizosphere microbiome at 7, 24, and 45 days post-treatment. Treatments included Control, Str only, SynCom only, All treatments, Str + pH, and Str + SynCom. Bars represent the mean relative abundance across replicates. Phyla such as Actinomycetota*,* Pseudomonadota*,* Chloroflexota, and Bacillota, and genera such as *Pseudomonas*, *Rhizobium*, and *Streptomyces* were among the most abundant. The data are presented as the mean of five independent replicates per treatment

Given that the SynCom was composed of *Pseudomonas*, we systematically investigated alterations in *Pseudomonas* and other predominant genera to assess SynCom establishment and its impact on community reassembly following streptomycin-induced dysbiosis. The composition and diversity of rhizosphere bacterial populations were affected by SynCom and streptomycin treatments in a time-dependent manner (Fig. 2 and Fig. S2). At the phylum level, Actinomycetota, Pseudomonadota, Chloroflexota, and Bacillota were predominant across all treatments and periods (Fig. 2A). No statistically significant differences were detected at the phylum level at Day 24. By Day 45, DESeq2 analysis revealed a statistically significant treatment-related alteration in Chloroflexota abundance (*P* < 0.05); however, this finding was not corroborated by pairwise comparisons. Pseudomonadota decreased in relative abundance (17.35% on average) when the SynCom was added under stress conditions (Str + pH + SynCom compared with Str + pH) at Day 24 (Fig. 2A), although this change did not reach statistical significance. *Pseudomonas*, *Rhizobium*, *Streptomyces*, and *Agrobacterium* ranked among the most prevalent taxa at the genus level. At Day 24, *Pseudomonas* relative abundance was significantly different among treatments (*P* < 0.05) and exhibited a modest increase in relative abundance in the Str + pH + SynCom treatment compared with Str + pH. The nonparametric Wald test did not identify significant pairwise differences (Table S3). By Day 45, DESeq2 analysis identified statistically significant variation in *Rhizobium* abundance (*P* < 0.05). The Str + pH + SynCom treatment group showed a lower abundance of *Rhizobium* compared with the Str + pH treatment group. In contrast, *Pseudomonas* and *Streptomyces* exhibited slight, non-significant increases in relative abundance; however, these trends were not supported by pairwise comparisons (Table S4). Overall, changes in *Pseudomonas* abundance were variable across time points and treatments and did not show a consistent increase following SynCom inoculation. Because taxonomic assignments were based on 16S rRNA gene sequencing, it was not possible to distinguish introduced SynCom strains from native populations; therefore, these results may reflect community-level shifts rather than direct evidence of SynCom strains’ establishment or persistence in the rhizosphere.

To determine whether these genus-level shifts were accompanied by broader changes in the rhizosphere microbiome, we analyzed community structure and alpha diversity across treatments and time points (Fig. S2). Principal component analysis (PCA) was performed on transformed relative abundance data to visualize patterns in community composition, revealing that the microbial community shifted over time, with partial separation among treatments at Days 7 and 24 and clearer divergence by Day 45 (Fig. S2A). Bray–Curtis dissimilarities were used for distance-based statistical analysis (PERMANOVA), which showed that composition was not significantly different among treatments at Day 7 (*P* = 0.228) or Day 24 (*P* = 0.229). However, a statistically significant effect was detected at Day 45 (*P* = 0.039), suggesting that treatment effects became detectable over time. This pattern suggests a gradual shift in microbial community structure rather than an immediate response to treatment. No significant differences in Shannon diversity were detected among treatments at any time point (Fig. S2B; Day 7: *P* = 0.34; Day 24: *P* = 0.49; Day 45: *P* = 0.47), suggesting that the treatments primarily influenced community composition rather than overall diversity.

### Effects of SynCom and pH on leaf nutrient rates of plants under dysbiosis

**Fig. 3:**
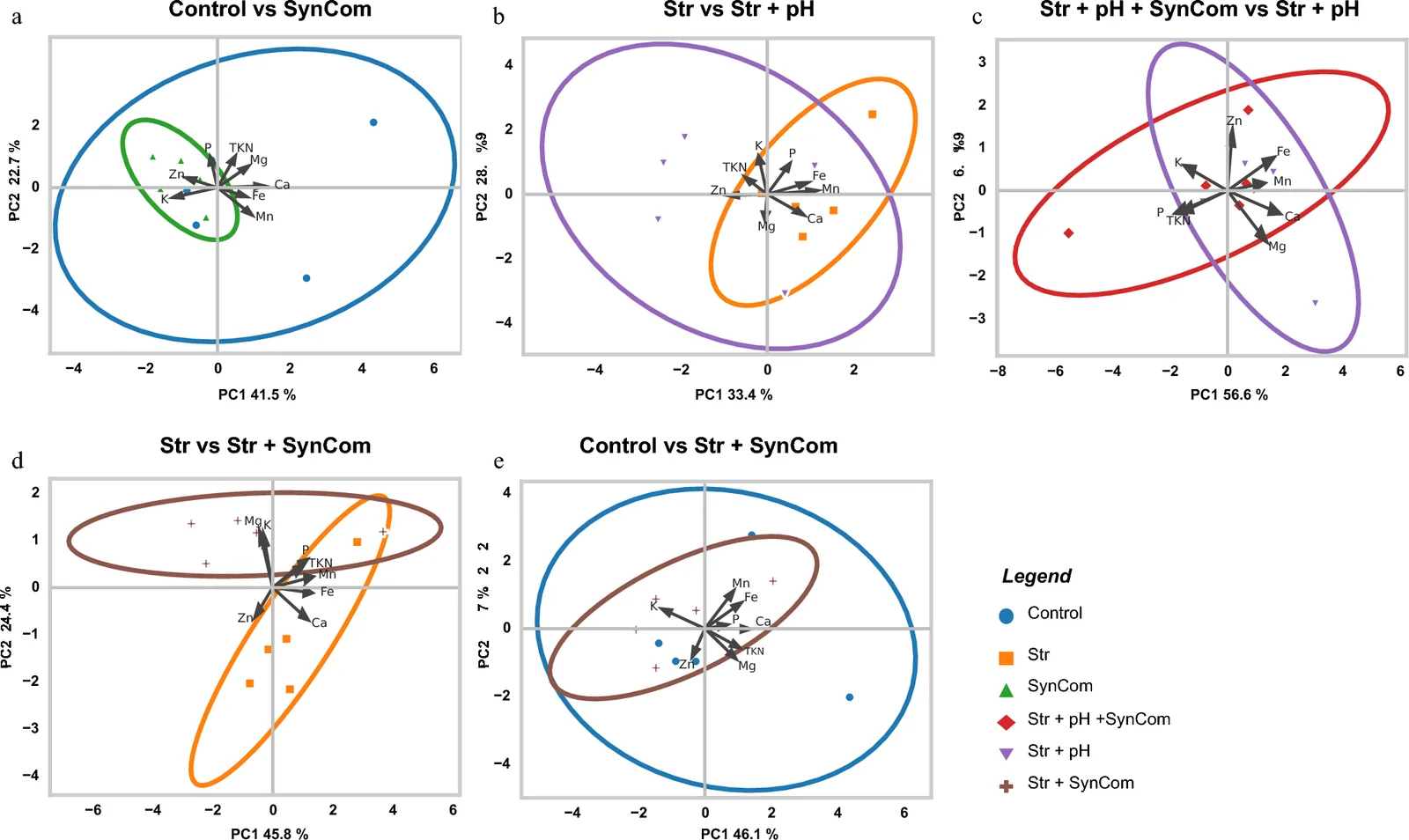
SynCom treatment alters leaf nutrients levels in multivariate space. Principal component analysis (PCA) of leaf nutrient concentrations revealed that plants treated with the SynCom have a nutrient profile shifted from both control and non-inoculated treatment groups. Leaves treated with SynCom reliably segregate along primary components influenced by variations in calcium (Ca), magnesium (Mg), iron (Fe), and manganese (Mn), indicating a slight but not significant alterations in these nutrients. Potassium (K), total nitrogen (TKN), and phosphorus (P) contribute to the distinction to a lesser extent, but zinc (Zn) exhibits minimal consistent differentiation among treatments. The data are presented as the mean of five independent replicates per treatment

**Fig. 4:**
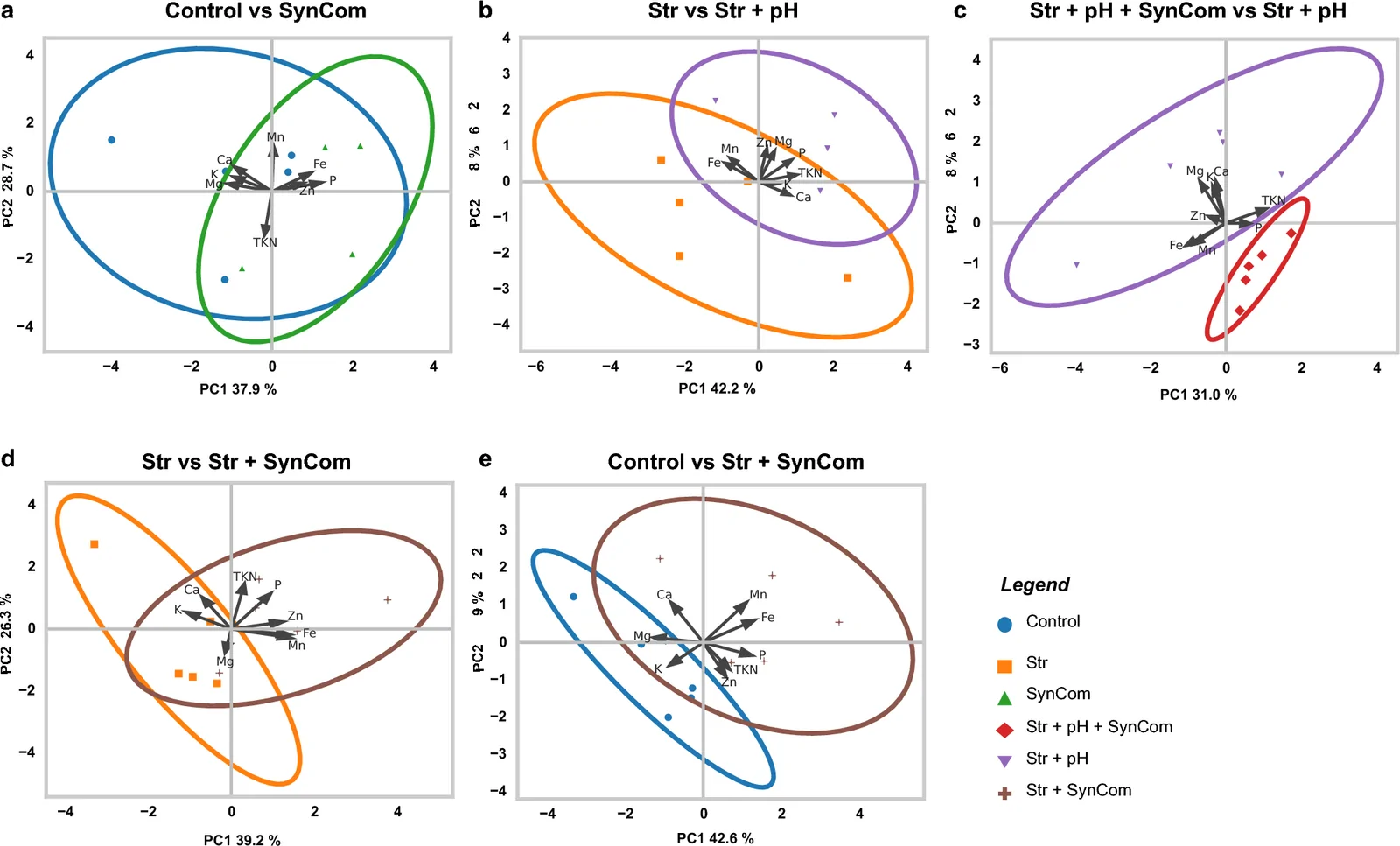
SynCom treatment reshapes root nutrient composition in multivariate space. Principal component analysis (PCA) of root nutrient concentrations showed that plants treated with SynCom develop a distinct root nutrient profile compared to controls and other treatments without SynCom. SynCom-treated roots consistently separate along principal components dominated by variation in calcium (Ca), magnesium (Mg), iron (Fe), and manganese (Mn), indicating strong and coordinated shifts in these nutrients. Potassium (K), total nitrogen (TKN), and phosphorus (P) contribute more modestly to the multivariate separation, whereas zinc (Zn) shows little consistent discrimination among treatments. The data are expressed as the mean of five independent replicates for each treatment

To determine whether microbiome disruption and restoration influenced plant nutritional status, we evaluated macro- and micronutrient concentrations in citrus leaves and roots, as these parameters reflect plant nutrient dynamics and overall physiological performance. At 45 days post-treatment, key macro- and micronutrient concentrations in citrus leaves varied significantly across treatments (Fig. 3). Multivariate analysis of leaf nutrient concentrations indicated that SynCom inoculation altered leaf nutrient composition in a treatment-specific manner. In the PCA space depicted in Fig. 3, SynCom-treated plants showed a tendency toward separation from non-SynCom-treated and control treatments, although partial overlap among treatments was observed, suggesting that the synthetic community modified the leaf nutrient composition. The separation was primarily influenced by variations in calcium (Ca), magnesium (Mg), iron (Fe), and manganese (Mn), which exhibited significant loadings on the primary components that differentiated SynCom from other groups in Fig. 3 A, C, D, and E. Box plot comparisons (Fig. S3) indicated that SynCom-treated plants displayed differences in Ca and Mg concentrations and changes in Fe and Mn distribution relative to non-SynCom-treated plants, consistent with a reorganization of leaf nutrient homeostasis. In contrast, adding the pH treatment to plants under Str-induced dysbiosis increased variation (Fig. 3B). Macronutrients, including potassium (K), total Kjeldahl nitrogen (TKN), and phosphorus (P), influenced the overall multivariate structure depicted in Fig. 3; nevertheless, they did not independently explain the significant differentiation of SynCom samples from the other treatments. Notably, SynCom-treated plants under stress exhibited a significant increase in leaf potassium concentration (25%) compared to stressed controls (Fig. S3), highlighting a shift in macronutrient allocation. Zinc (Zn) demonstrated the lowest and most variable correlation with SynCom, exhibiting considerable overlap between SynCom and non-SynCom leaves in the paired plots. The patterns illustrated in Figs. 4 and S4 suggest that SynCom influenced the relative balance of key cations and micronutrients in leaves with consistent contributions from Ca, Mg, Fe, and Mn to treatment separation.

### Effects of SynCom and pH on root nutrient rates of plants under dysbiosis

A simultaneous multivariate analysis of root nutrient content indicated that SynCom similarly created a partially distinct nutritional signature in the roots. Roots treated with SynCom formed a distinct cluster in PCA space compared to non-SynCom-treated and control treatments (Fig. 4), indicating that the microbial community modified root nutrient profiles at the whole-ionome level. The differentiation of SynCom roots, similar to leaves, was predominantly influenced by Ca, Mg, Fe, and Mn, which significantly contributed to the primary components that differentiated SynCom from other groups in Fig. 4A, C, D, and E. In paired nutrient plots (Fig. S4), roots treated with SynCom during dysbiosis exhibited reduced Ca and Mg concentrations, along with changes in Fe and Mn distribution, indicating a reorganization of micronutrient acquisition or storage at the root–soil interface. In the comparison of the Str treatment with the Str + pH group (Fig. 4B), there was a shift in root nutrient composition in dysbiotic roots with the addition of pH stress. Significantly, root potassium concentrations diminished by roughly 29% after SynCom treatment compared to stressed plants lacking SynCom (Fig. S4), in striking contrast to the rise noted in leaf tissues and suggesting coordinated alterations in nutrient distribution across plant tissues. Potassium (K), total Kjeldahl nitrogen (TKN), and phosphorus (P) contributed minimally to differentiating SynCom from other treatments in roots, as illustrated in Fig. 4, indicating that SynCom does not function only as a macronutrient fertilizer in the rhizosphere. Zn exhibited restricted and inconsistent separation in root tissues, likely reflecting the high variability in Zn concentrations observed across treatments in leaf tissues. The foundational results support the notion that SynCom predominantly alters cation and micronutrient homeostasis, consistently highlighting Ca, Mg, Fe, and Mn. Alongside the concomitant rise in leaf potassium, these findings imply synchronized alterations in nutrient allocation between belowground and aerial tissues.

## Discussion

In our previous study (Ketehouli et al. 2024), we demonstrated that treatments with streptomycin and oxytetracycline impaired citrus photosynthesis and modified rhizosphere bacterial composition, mainly the relative abundances of *Pseudomonas* and *Streptomyces*, under greenhouse conditions. Certain *Pseudomonas* and *Streptomyces* spp. are recognized for their ecological adaptability and beneficial characteristics for plants, making them suitable candidates for biocontrol and bioremediation applications in agroecosystems (Haas and Défago 2005a; Awasthi et al. 2015; Viaene et al. 2016). *Pseudomonas* spp. in the rhizosphere participate in intricate interactions with plant hosts and other soil microbes and can be effective biocontrol or bioremediation agents. *Pseudomonas* spp. use various mechanisms in their biocontrol role, including the synthesis of antibiotics (such as phenazines, pyoluteorin, 2,4-diacetylphloroglucinol), hydrogen cyanide, and siderophores, which inhibit soilborne pathogens and reduce nutrient competition (Haas and Défago 2005a; Awasthi et al. 2015; Santoyo et al. 2021). In this study, a SynCom composed of *Pseudomonas* spp. was designed and we investigated its effect on the bacterial composition in the rhizosphere and plant nutrient content when exposed to dysbiosis due to streptomycin and acidic stresses.

Genomic characterization and biosynthetic capabilities of the four *Pseudomonas* isolates collected from organic citrus rhizospheres clarified their phylogenetic placement within the genus and suggested that they represent genomically distinct lineages, while also revealing key metabolic features relevant to SynCom functionality. Phylogenomic investigations utilizing ANIb and dDDH d4 comparisons distinctly identified SMN1, SMN5, SMK4, and SMV3 as representatives of four distinct *Pseudomonas* spp, phylogenetically proximate but distinct from *P. vlassakiae*, *P. zarinae*, *P. izuensis*, and *P. fulva*, respectively. The antiSMASH analysis of biosynthetic gene clusters uncovered both conserved and strain-specific secondary metabolite pathways. A hydrogen cyanide (HCN) gene cluster in all isolates suggests a standard cyanogenic capability, which may help suppress the pathogen. The distinct duplication of HCN in SMN5 and the exclusive occurrence of Ririwpeptide ABC and ranthipeptide clusters in SMN1 and SMN5 highlight potential strain-specific functions in inter-microbial competition or plant–microbe signaling. The simultaneous presence of Stechlisins B2-F-like NRPS clusters and carotenoid-related terpene genes in SMV3 and SMK4 indicates metabolite convergence within this subclade, potentially affecting niche adaptation or rhizosphere persistence. The genetic characteristics and metabolic capabilities encoded by the SynCom strains could influence their ecological behavior, including root colonization, microbial interactions, and modification of the host plant environment. While genomic analysis found biosynthetic gene clusters possibly linked to plant-beneficial processes, such as hydrogen cyanide generation, these predictions were not empirically corroborated in this work. Similarly, essential properties of plant growth-promoting rhizobacteria (PGPR), including nitrogen fixation, phosphate solubilization, indole−3-acetic acid synthesis, and siderophore production, were not explicitly measured. Consequently, any suggested functional roles of SynCom members should be viewed with skepticism and considered hypotheses requiring experimental confirmation in subsequent research.

The analyses of community composition revealed that SynCom treatments were associated with shifts in the relative abundance of several genera, including *Pseudomonas* and *Streptomyces,* which are commonly reported to include plant-associated beneficial species. However, these changes were not consistent across treatments and should be interpreted as compositional trends rather than definitive enrichment. The observed increase in *Pseudomonas* and reduction of *Rhizobium* under SynCom treatments may suggest potential changes in microbial functional potential, including processes related to auxin (indole−3-acetic acid; IAA) production, ethylene modulation through ACC deaminase activity, or nutrient cycling (Masson-Boivin and Sachs 2018), although these functions were not directly evaluated in this study. *Pseudomonas* enrichment was predominantly identified by model-based DESeq2 analysis, which accounts for count structure and biological variability, whereas nonparametric Kruskal–Wallis tests did not consistently reveal statistically significant pairwise differences. Such discrepancies are not unexpected, as DESeq2 is more sensitive to subtle but consistent shifts in abundance when variance and sequencing depth are explicitly modeled, while rank-based approaches are inherently more conservative and less sensitive to modest effect sizes (Weiss et al. 2017; Lin and Peddada 2020). Thus, the detected changes in *Pseudomonas* likely reflect biologically meaningful but quantitatively modest responses to SynCom addition under dysbiotic conditions.

The principal component analysis and alpha diversity measurements demonstrated that microbial community responses were both treatment- and time-dependent. Although PCA (Fig. S2A) revealed temporal shifts in community composition, statistical tests did not support significant differentiation among treatments at any sampling point (Day 7: *P* = 0.34; Day 24: *P* = 0.49; Day 45: *P* = 0.47). These non-significant results suggest that, while microbial structure may trend differently over time depending on treatment, the overall divergence was not sufficiently robust to be distinguished statistically in our dataset. Comparable observations have been reported in other plant microbiome studies, where disturbance and inoculation effects yield subtle compositional trends that do not always translate into strong PERMANOVA separation, especially when within-group variability is high (Hartman et al. 2018). Time-dependent yet statistically variable changes are increasingly recognized as typical of rhizosphere microbiomes, where significant functional redundancy and robust environmental filtering can buffer communities against substantial compositional alterations following disturbance or inoculation (Louca et al. 2018). Temporal alterations in rhizosphere community composition indicate that SynCom treatment affects microbiome assembly under stress conditions. While SynCom application altered rhizosphere bacterial community composition over time, the stability of individual SynCom strains could not be evaluated due to the lack of resolution in the amplicon analysis utilized. In natural soil systems, imported microbial consortia often exhibit dynamic, temporary establishment due to competition with native microorganisms and environmental filtering (Zegeye et al. 2019; Velte et al. 2025). Thus, our results may indicate transient functional interactions rather than enduring long-term colonization, as the persistence and establishment of individual SynCom strains were not directly assessed. The 16S rRNA amplicon sequencing approach used in this study did not provide sufficient taxonomic resolution to distinguish introduced strains from indigenous Pseudomonas populations. Consequently, we cannot conclusively determine SynCom stability over time. Future studies should incorporate strain-resolved monitoring approaches, including strain-specific qPCR assays, fluorescent reporter systems, or genomic barcoding strategies, to quantify SynCom persistence and colonization dynamics under stress conditions. Moreover, imaging techniques such as fluorescence tagging or fluorescence in situ hybridization were not used in the current investigation. These methods may yield significant spatial insights into root colonization patterns and SynCom dispersion inside the rhizosphere. Nonetheless, differentiating transplanted SynCom strains from indigenous Pseudomonas populations necessitates strain-specific markers or probes, which were not addressed in the present study. Integrating these methodologies in forthcoming research might enhance our comprehension of SynCom spatial structure and plant–microbe interactions.

Furthermore, Shannon diversity indices showed no statistically significant variation across treatments and time points, indicating that the richness and evenness of bacterial communities remained stable under both streptomycin disruption and SynCom application, despite fluctuations in relative abundances. The dissociation of taxonomic composition from alpha diversity observed here indicates that SynCom-induced impacts may function through compositional rebalancing among predominant phyla and genera rather than through increases or decreases in overall diversity. These findings thus indicate that targeted microbial interventions may direct rhizosphere communities toward favorable structures during stress, while preserving broad community stability.

In addition to modulating rhizosphere microbial communities, SynCom treatment influenced nutrient concentrations and altered root–leaf translocation. The *Pseudomonas*-based SynCom altered the mineral nutrient profiles of both roots and leaves in plants subjected to streptomycin (Str) and low-pH conditions. The most pronounced effects were observed in iron (Fe) and manganese (Mn), with notable shifts in calcium (Ca), magnesium (Mg), potassium (K), zinc (Zn), phosphorus (P), and total Kjeldahl nitrogen (TKN). These minerals are essential for citrus growth, as citrus species are especially susceptible to micronutrient imbalances and phosphorus deficiencies, which directly influence photosynthesis, root formation, and nutrient transport (Huang et al. 2021; Meng et al. 2021). Importantly, the strains included in this SynCom were selected for their compatibility, genomic features, and performance under streptomycin-induced dysbiosis and low-pH conditions. However, their tolerance to other abiotic stresses was not evaluated in this study. This represents an important limitation, as strain performance may vary under different environmental contexts. However, the addition of SynCom did not fully restore the nutrient profile to that of the stressed plant group. This indicates a partial physiological rescue, in which microbial functions may support nutrient dynamics, including uptake, redistribution, or homeostasis, during stress rather than fully restoring plant nutrient status. These patterns are consistent with known functional traits of *Pseudomonas* spp. involved in nutrient mobilization and plant–microbe signaling. The mechanisms by which *Pseudomonas* spp. modify plant nutrient profiles have been previously described and align with the principal component analysis (PCA) results. For instance, fluorescent *Pseudomonas* produce high-affinity siderophores, such as pyoverdine, that chelate Fe(III) and make it available to plants, thereby potentially influencing Fe availability under restrictive conditions (Cézard et al. 2015). These siderophore biosynthetic pathways (pyoverdine gene clusters) are well characterized and contribute to competitive iron acquisition in the rhizosphere (Lamont and Martin 2003; Moon et al. 2008). Iron is crucial for chlorophyll synthesis and electron transport in citrus foliage, and a deficiency of Fe swiftly results in interveinal chlorosis and diminished photosynthetic efficiency (Jin et al. 2017). The increased availability of Fe often leads to co-regulation with Mn (which is integral to the oxygen-evolving complex of photosystem II and is hence closely associated with photochemical efficiency in citrus (Tang et al. 2012) in photosynthetic tissues, consistent with the PCA results showing strong Fe/Mn loadings that differentiate Str + pH from All treatments group in leaves. These effects are likely amplified at low pH, where the solubility of Fe and Mn is decreased. Under such conditions, SynCom may contribute to Fe/Mn modulation, either through effects on availability or through plant regulation, rather than directly increasing uptake. The dominance of Fe and Mn on positive PCA axes in leaves contrasts aligns with siderophore-mediated iron handling and associated manganese co-regulation in the presence of SynCom (Haas and Défago 2005b; Crowley 2006; Mažylytė et al. 2024).

In roots, the patterns of phosphorus and zinc further support SynCom’s role in facilitating nutrient access. Phosphorus is essential for citrus root development, ATP production, and sucrose transfer; its deficiency can significantly impede nitrogen utilization efficiency and vegetative growth (Meng et al. 2021). Many *Pseudomonas* strains can solubilize inorganic phosphorus through organic acid production and phosphatase activity, thereby increasing the availability of orthophosphate in the rhizosphere. *Pseudomonas* spp. not only acquire iron but also affect plant nutrient dynamics through various mechanisms, including phosphate solubilization via organic acid production (gluconic acid through pyrroloquinoline quinone-dependent pathways) and the modulation of root system architecture through phytohormone production, such as indole−3-acetic acid (IAA), which can increase root surface area and potentially influence nutrient acquisition capacity (Anthony 2001; Duca et al. 2014). Changes in phosphorus observed in the All treatments group compared with the Str+pH group may contribute to shifts in root nutrient composition and nitrogen-related parameters, as reflected in the root PCA, where P and TKN contribute to separation (Fig. 4C). The correlation between P and TKN is especially relevant for citrus, as synchronized P and N supply facilitates continuous canopy development and root metabolic function (Kaur et al. 2024). Zinc responses, however, are more variable; while *Pseudomonas* can mobilize Zn from insoluble complexes, Zn competes with Fe at transport interfaces and is sensitive to pH. Zinc is essential for enzyme activation, auxin metabolism, and leaf development in citrus; however, both excess and deficiency can disturb iron homeostasis (Srivastava and Singh 2005; Rai et al. 2021). Our results indicate that Zn contributed to separation in some comparisons, suggesting that SynCom did not universally restore Zn to control levels. This pattern is consistent with a SynCom that is effective for Fe/Mn and P but less uniformly effective for Zn under acidic and antibiotic stress (Richardson et al. 2009; Pattnaik et al. 2021).

The observed increase in leaf potassium and decrease in root potassium under SynCom treatment suggest changes in nutrient distribution rather than enhanced uptake alone. However, these observations remain correlative and should not be interpreted as direct evidence of SynCom-mediated nutrient regulation. Because nutrient uptake rates, transporter activities, and nutrient fluxes were not measured, the underlying mechanisms cannot be conclusively established. It is possible that microbial interactions indirectly influenced plant nutrient partitioning under combined stress conditions. Future studies integrating isotope tracing, nutrient flux measurements, and analyses of nutrient transporter activity will be necessary to determine whether SynCom directly modulates potassium allocation and broader nutrient homeostasis. Therefore, these interpretations should be considered as biologically plausible hypotheses rather than demonstrated mechanisms.

The current study did not examine molecular plant responses. Transcriptomic or targeted gene expression analyses of nutrient transporters, stress-responsive pathways, and plant signaling networks would significantly enhance mechanistic understanding of SynCom-mediated effects. Our work focused primarily on physiological, dietary, and microbial responses, leaving the molecular pathways that underlie these responses unaddressed. Future investigations that integrate transcriptomics and metabolomics will be crucial for clarifying the pathways via which SynCom affects plant response to simultaneous abiotic and antibiotic stressors.

We did not use a pH-only treatment, which limits the ability to isolate the independent effect of low pH from its interactions with streptomycin and SynCom application. The induced low pH (3.5) is a major perturbation that may affect both plant physiology and microbial community composition, potentially interacting with antibiotic-induced dysbiosis. Thus, the observed reactions ought to be considered a consequence of synergistic stress rather than the effects of individual factors. Subsequent research with a comprehensive factorial design, incorporating pH-only treatments, would provide the formal examination of main effects and interactions between stressors and microbial inoculation.

## Conclusion and perspectives

This study used four *Pseudomonas* isolates from citrus soil, representing taxonomic and potential functional diversity, to assemble a synthetic microbial community (SynCom) and examined their impact on citrus rhizosphere microbiome dynamics under abiotic and chemical stresses. The SynCom was associated with changes in the rhizosphere bacterial composition and altered foliar and root nutrient dynamics, especially potassium translocation, under streptomycin and low-pH stress conditions. However, direct persistence of the introduced SynCom strains, functional plant growth-promoting rhizobacteria (PGPR) activities, and the molecular mechanisms underlying these responses were not assessed and therefore cannot be conclusively inferred from the present study. These findings highlighted the potential of *Pseudomonas*-based SynCom as a promising biotechnological approach to support nutrient homeostasis and microbiome resilience under stressed agricultural environments, while emphasizing the need for further mechanistic investigations. Future studies should also assess the consistency and stability of SynCom effects at greenhouse and field scales across various soil types, crop species, and environmental conditions. Integrating transcriptomics, metabolomics, and ionomics will enhance our awareness of the impact of SynComs on host physiology at the cellular level. Although this work was carried out under controlled growth chamber conditions, a field experiment could yield a more realistic outcome, given the more complex system in the field: native microbiomes, variable soils, changing abiotic conditions, etc. Translating SynCom-based approaches into sustainable crop management strategies will require bridging the gap between controlled microbiome engineering and field-scale validation. Utilizing microbial consortia designed for specific plant and soil environments may enhance microbiome-informed approaches for sustainable agricultural production alongside rising environmental stress.

## Supplementary Information

Below is the link to the electronic supplementary material.

Supplementary file1 (DOCX 5732 KB)

## Data Availability

The datasets analyzed in the current study are openly available in the GenBank repository under PRJNA1280539: https://www.ncbi.nlm.nih.gov/bioproject/PRJNA1280539/. This published article and its supplementary information files include all other data generated or analyzed during this study.

## Abbreviations

ANIb: Average nucleotide identity (BLAST-based)
BGCs: Biosynthetic gene clusters
DESeq2: Differential gene expression analysis based on the negative binomial distribution
dDDH d4: Digital DNA–DNA hybridization (formula d4)
NRPS: Non-ribosomal peptide synthetase
PERMANOVA: Permutational multivariate analysis of variance
SynCom: Synthetic microbial community
