# Supplementary material for: Pseudomonas spp.-based synthetic microbial community effects on nutrient dynamics in citrus plants under induced dysbiosis
Source: Planta. 2026 Oct 29;264(6):181. doi: 10.1007/s00425-026-05205-6 (PMC13719819; doi:10.1007/s00425-026-05205-6)
Supplement: Supplementary file 1 [file 425_2026_5205_MOESM1_ESM.docx]

**Supplementary data**

***Pseudomonas* spp.-based synthetic microbial community effects on nutrient uptake in citrus plants under induced dysbiosis**

Toi Ketehouli^1^, Mason R. Trub^2^, Erica M. Goss^1,3^, Samuel J. Martins^1*^

^1^ Department of Plant Pathology, Institute of Food and Agricultural Sciences, University of Florida, Gainesville, FL, 32611, USA

^2^ Department of Microbiology and Cell Science, University of Florida, Gainesville, FL, 32603, USA

^3^ Emerging Pathogens Institute, University of Florida, Gainesville, FL, 32610, USA

**Fig. S1** Growth of selected *Pseudomonas* isolates (SMN1, SMN5, SMK4, and SMV3) under different pH conditions was assessed via absorbance at OD_655_ using a microplate reader. Under acidic conditions (pH 3.5), SMN1 and SMN5 exhibited relatively higher growth than SMK4 and SMV3, indicating better acid tolerance than other isolates. Bars represent mean absorbance values with error bars showing standard deviation across five biological replicates. Different letters above bars indicate statistically significant differences among isolates (*P* < 0.05). In bold are the four *Pseudomonas* isolates used to design the synthetic microbial community (SynCom). Each graph displays nutrient concentration (mg g⁻¹ leaf tissue) ± SE of five biological replications.

**Fig. S2 Microbial community structure and alpha diversity under different treatments and time points. A** Principal Component Analysis (PCA) plots of microbial community composition at Days 7, 24, and 45 post-first pH treatment based on Bray–Curtis distances. Each dot represents a biological replicate, and clustering reflects treatment effects on community structure. The percentage of explained variance is shown for each dimension. **B** Shannon diversity indices of bacterial communities across treatments and time points. Statistical comparisons were performed to assess differences in community diversity. Although temporal patterns are evident, most comparisons were not statistically significant (*P* > 0.05), except at Day 45 (*P* = 0.039), where differences in diversity were observed.

**Fig. S3** Nutrient concentrations in plant leaves under different treatment conditions. **A** Total Kjeldahl nitrogen (TKN) concentrations. **B** Phosphorus (P) concentrations. **C** Potassium (K) concentrations. **D** Calcium (Ca) concentrations. **E** Iron (Fe) concentrations. **F** Magnesium (Mg) concentrations. **G** Manganese (Mn) concentrations. **H** Zinc (Zn) concentrations. Data are presented as boxplots (median, interquartile range, and outliers), with different letters indicating statistically significant differences among treatments. Notably, the Str + pH + SynCom treatment shows elevated potassium levels (**C**) and increased relative concentrations of most nutrients. Each graph displays nutrient concentration (mg g⁻¹ leaf tissue) ± SE of five biological replications.

**Fig. S4** Nutrient concentrations in plant roots under different treatment conditions. **A** Total Kjeldahl nitrogen (TKN) rate. **B** Phosphorus (P). **C** Iron (Fe) concentrations. **D** Potassium (K) concentrations. **E** Calcium (Ca) concentrations. **F** Magnesium (Mg) concentrations. **G** Zinc (Zn) concentrations. **H** Manganese (Mn) concentrations in roots. Across panels, treatments differentially affected root nutrient status, with the Str + pH + SynCom treatment generally associated with reduced concentrations of several nutrients. Each panel shows mean nutrient concentration (mg g⁻¹ root tissue) ± SE of five biological replications.

**Fig. S5** Average Nucleotide Identity based on BLAST **(**ANIb) alignment coverage among four bacterial isolates (SMN1, SMN5, SMK4, and SMV3). The heatmap shows pairwise genome alignment coverage percentages for each isolates, with rows and columns representing each isolate. Darker shading indicates higher alignment coverage, and numerical values within each cell report the exact percentage of genome coverage for that comparison. Diagonal cells are 100%, reflecting self-comparisons. All pairwise ANIb values fall below commonly used species-level thresholds (80-94% ANI), indicating that each isolate represents a distinct genomospecies, while still belonging to the same genus.

**Fig. S6 Total mapped read counts across treatment groups.** Barplot showing the total number of quality-filtered and mapped 16S rRNA gene sequence reads per treatment group. While most samples exhibited comparable sequencing depth, some variation in library size was observed. These differences were accounted for in downstream analyses to minimize potential bias from sequencing depth.

**Table S1 Experimental schedule of applications and sampling during the 45-day study.**

| **Days** | **Activities** |
| --- | --- |
| Day1 | pH application |
| Day4 | pH application |
| Day7 | **First day of sampling** and pH application |
| Day8 | Str application |
| Day9 | SynCom application |
| Day14 | pH application |
| Day15 | Str application |
| Day16 | SynCom application |
| Day21 | pH application |
| Day22 | Str application |
| Day23 | SynCom application |
| Day24 | **Second day of sampling** |
| Day28 | pH application |
| Day29 | Str application |
| Day30 | SynCom application |
| Day35 | pH application |
| Day36 | Str application |
| Day37 | SynCom application |
| Day42 | pH application |
| Day43 | Str application |
| Day44 | SynCom application |
| Day45 | **Last day of sampling** |

**Table S2** Interspecies interaction matrix of 13 *Pseudomonas isolates*. Interactions were assessed based on pairwise co-culturing on King's B solid medium. (+) indicates a positive or compatible interaction between two isolates, while (–) denotes no visible growth or inhibition. The diagonal squares represent self-comparisons.

| **Isolates** | **SMV3** | SMB8 | SMV7 | SMB3 | SMB4 | **SMN1** | SMV71 | **SMK4** | SMN12 | SMV4 | SMK5 | **SMN5** | SMP6 |
| --- | --- | --- | --- | --- | --- | --- | --- | --- | --- | --- | --- | --- | --- |
| **SMV3** |  | - | + | + | - | + | + | + | + | - | - | + | + |
| SMB8 | - |  | + | + | + | + | + | + | + | + | + | + | + |
| SMV7 | + | + |  | + | + | + | + | + | + | + | + | + | + |
| SMB3 | + | + | + |  | + | + | + | + | + | + | - | + | + |
| SMB4 | - | + | + | + |  | - | + | + | + | + | + | + | + |
| **SMN1** | + | + | + | + | - |  | + | + | + | - | - | + | + |
| SMV71 | + | + | + | + | + | + |  | + | + | + | + | + | + |
| **SMK4** | + | + | + | + | + | + | + |  | + | + | - | + | + |
| SMN12 | + | + | + | + | + | + | + | + |  | + | + | + | + |
| SMV4 | - | + | + | + | + | - | + | + | + |  | + | + | + |
| SMK5 | - | + | + | - | + | - | + | - | + | + |  | + | + |
| **SMN5** | + | + | + | + | + | + | + | + | + | + | + |  | + |
| SMP6 | + | + | + | + | + | + | + | + | + | + | + | + |  |

**Table S3** DESeq2-based pairwise comparisons of differentially abundant bacterial genera across treatments at day 24. Results include log2 fold change estimates, Wald test statistics, raw *P*-values, and Benjamini–Hochberg adjusted *P*-values (*P*adj). Analyses were performed on normalized count data, and significant differences (*P*adj < 0.05) are highlighted.

| **Genus** | **baseMean** | **log2FoldChange** | **lfcSE** | **stat** | ***P*value** | ***P*adj** | **contrast** | **Day** |
| --- | --- | --- | --- | --- | --- | --- | --- | --- |
| Agrobacterium | 12358.4689 | -0.9497503 | 0.52235652 | -1.8182032 | 0.06903308 | 0.13806617 | Ctrl vs All Treatment | Day24 |
| Pseudomonas | 3226.20913 | -0.6215233 | 0.88192445 | -0.7047354 | 0.48097495 | 0.64129993 | Ctrl vs All Treatment | Day24 |
| Rhizobium | 3016.183 | -0.0656739 | 0.56873697 | -0.1154732 | 0.90807009 | 0.90807009 | Ctrl vs All Treatment | Day24 |
| Streptomyces | 2696.47535 | 1.44695731 | 0.45156961 | 3.20428406 | 0.00135399 | 0.00541595 | Ctrl vs All Treatment | Day24 |
| Agrobacterium | 12358.4689 | -0.7008682 | 0.52235679 | -1.3417422 | 0.17967959 | 0.1802143 | Ctrl vs Str | Day24 |
| Pseudomonas | 3226.20913 | -2.2223011 | 0.88187511 | -2.5199726 | 0.0117364 | 0.04694559 | Ctrl vs Str | Day24 |
| Rhizobium | 3016.183 | -0.8029956 | 0.56869792 | -1.4119897 | 0.157953 | 0.1802143 | Ctrl vs Str | Day24 |
| Streptomyces | 2696.47535 | 0.60497831 | 0.45144418 | 1.34009549 | 0.1802143 | 0.1802143 | Ctrl vs Str | Day24 |
| Agrobacterium | 12358.4689 | -1.9854929 | 0.5223497 | -3.8010798 | 0.00014407 | 0.00057627 | Ctrl vs Str+pH | Day24 |
| Pseudomonas | 3226.20913 | -0.7955136 | 0.88194492 | -0.9019992 | 0.36705728 | 0.36705728 | Ctrl vs Str+pH | Day24 |
| Rhizobium | 3016.183 | -0.6304189 | 0.56873619 | -1.1084557 | 0.26766507 | 0.35688676 | Ctrl vs Str+pH | Day24 |
| Streptomyces | 2696.47535 | 1.00382974 | 0.45158777 | 2.22288958 | 0.02622325 | 0.05244649 | Ctrl vs Str+pH | Day24 |
| Agrobacterium | 12358.4689 | -1.2517059 | 0.52235495 | -2.3962747 | 0.01656267 | 0.03312535 | Ctrl vs Str+SynCom | Day24 |
| Pseudomonas | 3226.20913 | -0.5580832 | 0.88193842 | -0.6327915 | 0.52686979 | 0.70249306 | Ctrl vs Str+SynCom | Day24 |
| Rhizobium | 3016.183 | -0.0367218 | 0.56875096 | -0.0645658 | 0.94851974 | 0.94851974 | Ctrl vs Str+SynCom | Day24 |
| Streptomyces | 2696.47535 | 1.38868741 | 0.45159498 | 3.07507277 | 0.00210451 | 0.00841805 | Ctrl vs Str+SynCom | Day24 |
| Agrobacterium | 12358.4689 | -0.6385707 | 0.52235611 | -1.2224815 | 0.22152561 | 0.47099975 | Ctrl vs SynCom | Day24 |
| Pseudomonas | 3226.20913 | 0.50142116 | 0.88197421 | 0.56852134 | 0.56968102 | 0.7595747 | Ctrl vs SynCom | Day24 |
| Rhizobium | 3016.183 | -0.0312276 | 0.56872192 | -0.0549085 | 0.95621139 | 0.95621139 | Ctrl vs SynCom | Day24 |
| Streptomyces | 2696.47535 | 0.53553754 | 0.45143134 | 1.18631006 | 0.23549988 | 0.47099975 | Ctrl vs SynCom | Day24 |
| Agrobacterium | 12358.4689 | 0.24888212 | 0.52229894 | 0.47651278 | 0.63370911 | 0.63370911 | All Treatment vs Str | Day24 |
| Pseudomonas | 3226.20913 | -1.6007778 | 0.88175795 | -1.815439 | 0.06945647 | 0.13891295 | All Treatment vs Str | Day24 |
| Rhizobium | 3016.183 | -0.7373217 | 0.56862947 | -1.2966647 | 0.19474659 | 0.25966212 | All Treatment vs Str | Day24 |
| Streptomyces | 2696.47535 | -0.841979 | 0.45152719 | -1.864736 | 0.06221842 | 0.13891295 | All Treatment vs Str | Day24 |
| Agrobacterium | 12358.4689 | -1.0357426 | 0.52229184 | -1.9830726 | 0.04735933 | 0.18943731 | All Treatment vs Str+pH | Day24 |
| Pseudomonas | 3226.20913 | -0.1739903 | 0.88182777 | -0.1973064 | 0.84358774 | 0.84358774 | All Treatment vs Str+pH | Day24 |
| Rhizobium | 3016.183 | -0.564745 | 0.56866774 | -0.9931019 | 0.32066031 | 0.43540086 | All Treatment vs Str+pH | Day24 |
| Streptomyces | 2696.47535 | -0.4431276 | 0.45167075 | -0.9810854 | 0.32655065 | 0.43540086 | All Treatment vs Str+pH | Day24 |
| Agrobacterium | 12358.4689 | -0.3019556 | 0.52229709 | -0.5781301 | 0.56317632 | 0.95939669 | All Treatment vs Str+SynCom | Day24 |
| Pseudomonas | 3226.20913 | 0.06344018 | 0.88182128 | 0.07194222 | 0.94264789 | 0.95939669 | All Treatment vs Str+SynCom | Day24 |
| Rhizobium | 3016.183 | 0.02895201 | 0.56868251 | 0.05091068 | 0.95939669 | 0.95939669 | All Treatment vs Str+SynCom | Day24 |
| Streptomyces | 2696.47535 | -0.0582699 | 0.45167796 | -0.1290076 | 0.89735163 | 0.95939669 | All Treatment vs Str+SynCom | Day24 |
| Agrobacterium | 12358.4689 | 0.31117962 | 0.52229825 | 0.59578912 | 0.55131611 | 0.73508815 | All Treatment vs SynCom | Day24 |
| Pseudomonas | 3226.20913 | 1.12294449 | 0.88185706 | 1.27338606 | 0.20288108 | 0.40576216 | All Treatment vs SynCom | Day24 |
| Rhizobium | 3016.183 | 0.03444621 | 0.56865347 | 0.06057505 | 0.95169764 | 0.95169764 | All Treatment vs SynCom | Day24 |
| Streptomyces | 2696.47535 | -0.9114198 | 0.45151436 | -2.0185842 | 0.04353045 | 0.1741218 | All Treatment vs SynCom | Day24 |
| Agrobacterium | 12358.4689 | -1.2846247 | 0.52229212 | -2.4595905 | 0.01390956 | 0.05563825 | Str vs Str+pH | Day24 |
| Pseudomonas | 3226.20913 | 1.42678747 | 0.88177842 | 1.61807938 | 0.10564549 | 0.21129099 | Str vs Str+pH | Day24 |
| Rhizobium | 3016.183 | 0.17257675 | 0.56862869 | 0.30349638 | 0.76151161 | 0.76151161 | Str vs Str+pH | Day24 |
| Streptomyces | 2696.47535 | 0.39885143 | 0.45154535 | 0.88330315 | 0.3770725 | 0.50276334 | Str vs Str+pH | Day24 |
| Agrobacterium | 12358.4689 | -0.5508378 | 0.52229737 | -1.054644 | 0.2915882 | 0.2915882 | Str vs Str+SynCom | Day24 |
| Pseudomonas | 3226.20913 | 1.66421795 | 0.88177193 | 1.88735647 | 0.0591124 | 0.1652735 | Str vs Str+SynCom | Day24 |
| Rhizobium | 3016.183 | 0.76627376 | 0.56864346 | 1.34754695 | 0.17780414 | 0.23707219 | Str vs Str+SynCom | Day24 |
| Streptomyces | 2696.47535 | 0.7837091 | 0.45155256 | 1.73558777 | 0.08263675 | 0.1652735 | Str vs Str+SynCom | Day24 |
| Agrobacterium | 12358.4689 | 0.0622975 | 0.52229853 | 0.11927565 | 0.90505697 | 0.90505697 | Str vs SynCom | Day24 |
| Pseudomonas | 3226.20913 | 2.72372226 | 0.88180772 | 3.08879387 | 0.00200971 | 0.00803883 | Str vs SynCom | Day24 |
| Rhizobium | 3016.183 | 0.77176796 | 0.56861441 | 1.35727822 | 0.17469283 | 0.34938565 | Str vs SynCom | Day24 |
| Streptomyces | 2696.47535 | -0.0694408 | 0.45138891 | -0.153838 | 0.87773747 | 0.90505697 | Str vs SynCom | Day24 |
| Agrobacterium | 12358.4689 | 0.73378697 | 0.52229027 | 1.4049409 | 0.16003885 | 0.52559817 | Str+pH vs Str+SynCom | Day24 |
| Pseudomonas | 3226.20913 | 0.23743047 | 0.88184174 | 0.26924386 | 0.78774203 | 0.78774203 | Str+pH vs Str+SynCom | Day24 |
| Rhizobium | 3016.183 | 0.59369701 | 0.56868173 | 1.0439882 | 0.29649085 | 0.52559817 | Str+pH vs Str+SynCom | Day24 |
| Streptomyces | 2696.47535 | 0.38485767 | 0.45169611 | 0.85202786 | 0.39419863 | 0.52559817 | Str+pH vs Str+SynCom | Day24 |
| Agrobacterium | 12358.4689 | 1.34692223 | 0.52229143 | 2.578871 | 0.00991238 | 0.03964952 | Str+pH vs SynCom | Day24 |
| Pseudomonas | 3226.20913 | 1.29693479 | 0.88187753 | 1.47065181 | 0.1413853 | 0.28277061 | Str+pH vs SynCom | Day24 |
| Rhizobium | 3016.183 | 0.59919121 | 0.56865268 | 1.0537033 | 0.29201878 | 0.29968118 | Str+pH vs SynCom | Day24 |
| Streptomyces | 2696.47535 | -0.4682922 | 0.45153251 | -1.0371173 | 0.29968118 | 0.29968118 | Str+pH vs SynCom | Day24 |
| Agrobacterium | 12358.4689 | 0.61313526 | 0.52229668 | 1.17392142 | 0.2404265 | 0.32056867 | Str+SynCom vs SynCom | Day24 |
| Pseudomonas | 3226.20913 | 1.05950431 | 0.88187104 | 1.20142772 | 0.22958533 | 0.32056867 | Str+SynCom vs SynCom | Day24 |
| Rhizobium | 3016.183 | 0.0054942 | 0.56866746 | 0.00966153 | 0.99229133 | 0.99229133 | Str+SynCom vs SynCom | Day24 |
| Streptomyces | 2696.47535 | -0.8531499 | 0.45153972 | -1.8894237 | 0.05883507 | 0.23534029 | Str+SynCom vs SynCom | Day24 |

**Table S4** DESeq2-based pairwise comparisons of differentially abundant bacterial genera across treatments at day 45. Results include log2 fold change estimates, Wald test statistics, raw *P*-values, and Benjamini–Hochberg adjusted *P*-values (*P*adj). Analyses were performed on normalized count data, and significant differences (*P*adj < 0.05) are highlighted.

| **Genus** | **baseMean** | **log2FoldChange** | **lfcSE** | **stat** | ***P*value** | ***P*adj** | **Comparison** | **Day** |
| --- | --- | --- | --- | --- | --- | --- | --- | --- |
| Agrobacterium | 11741.8041 | 0.2380073 | 0.57209307 | 0.41602899 | 0.67738879 | 0.84902309 | Ctrl vs All Treatment | Day45 |
| Pseudomonas | 3453.43072 | -1.5941832 | 0.80467573 | -1.9811499 | 0.04757447 | 0.19029788 | Ctrl vs All Treatment | Day45 |
| Rhizobium | 2128.74471 | 0.12819461 | 0.67341467 | 0.19036504 | 0.84902309 | 0.84902309 | Ctrl vs All Treatment | Day45 |
| Streptomyces | 3112.17965 | -0.2105685 | 0.45948809 | -0.4582675 | 0.64676024 | 0.84902309 | Ctrl vs All Treatment | Day45 |
| Agrobacterium | 11741.8041 | 0.3433168 | 0.57207196 | 0.6001287 | 0.54842047 | 0.75037129 | Ctrl vs Str | Day45 |
| Pseudomonas | 3453.43072 | 0.12631967 | 0.80485012 | 0.15694807 | 0.87528577 | 0.87528577 | Ctrl vs Str | Day45 |
| Rhizobium | 2128.74471 | -1.5913396 | 0.67290555 | -2.3648782 | 0.018036 | 0.07214401 | Ctrl vs Str | Day45 |
| Streptomyces | 3112.17965 | -0.2658453 | 0.45936815 | -0.5787195 | 0.56277847 | 0.75037129 | Ctrl vs Str | Day45 |
| Agrobacterium | 11741.8041 | -0.0177084 | 0.5720333 | -0.0309569 | 0.97530395 | 0.97858695 | Ctrl vs Str+pH | Day45 |
| Pseudomonas | 3453.43072 | -0.021598 | 0.80467917 | -0.0268405 | 0.97858695 | 0.97858695 | Ctrl vs Str+pH | Day45 |
| Rhizobium | 2128.74471 | -0.815993 | 0.67288441 | -1.2126793 | 0.22525247 | 0.90100989 | Ctrl vs Str+pH | Day45 |
| Streptomyces | 3112.17965 | 0.18472271 | 0.45928882 | 0.40219291 | 0.68754206 | 0.97858695 | Ctrl vs Str+pH | Day45 |
| Agrobacterium | 11741.8041 | 0.98606397 | 0.57205995 | 1.72370742 | 0.08476068 | 0.16952135 | Ctrl vs Str+SynCom | Day45 |
| Pseudomonas | 3453.43072 | -1.8423175 | 0.80458236 | -2.2897812 | 0.02203401 | 0.08813602 | Ctrl vs Str+SynCom | Day45 |
| Rhizobium | 2128.74471 | -0.3908275 | 0.67293166 | -0.5807834 | 0.56138646 | 0.56138646 | Ctrl vs Str+SynCom | Day45 |
| Streptomyces | 3112.17965 | 0.30380655 | 0.45931167 | 0.66143878 | 0.50833096 | 0.56138646 | Ctrl vs Str+SynCom | Day45 |
| Agrobacterium | 11741.8041 | 0.32204308 | 0.57204718 | 0.56296594 | 0.57345808 | 0.64666048 | Ctrl vs SynCom | Day45 |
| Pseudomonas | 3453.43072 | -1.1169458 | 0.80462164 | -1.3881627 | 0.1650875 | 0.64666048 | Ctrl vs SynCom | Day45 |
| Rhizobium | 2128.74471 | 0.30854546 | 0.67308278 | 0.45840642 | 0.64666048 | 0.64666048 | Ctrl vs SynCom | Day45 |
| Streptomyces | 3112.17965 | -0.2742234 | 0.4592731 | -0.5970813 | 0.55045312 | 0.64666048 | Ctrl vs SynCom | Day45 |
| Agrobacterium | 11741.8041 | 0.1053095 | 0.57211377 | 0.18407091 | 0.85395783 | 0.90424365 | All Treatment vs Str | Day45 |
| Pseudomonas | 3453.43072 | 1.72050289 | 0.8047438 | 2.13795109 | 0.03252071 | 0.06504143 | All Treatment vs Str | Day45 |
| Rhizobium | 2128.74471 | -1.7195343 | 0.67320054 | -2.5542675 | 0.01064115 | 0.04256459 | All Treatment vs Str | Day45 |
| Streptomyces | 3112.17965 | -0.0552768 | 0.45948248 | -0.1203023 | 0.90424365 | 0.90424365 | All Treatment vs Str | Day45 |
| Agrobacterium | 11741.8041 | -0.2557157 | 0.57207512 | -0.4469966 | 0.65487749 | 0.65487749 | All Treatment vs Str+pH | Day45 |
| Pseudomonas | 3453.43072 | 1.57258523 | 0.80457283 | 1.95455919 | 0.05063512 | 0.20254049 | All Treatment vs Str+pH | Day45 |
| Rhizobium | 2128.74471 | -0.9441876 | 0.67317941 | -1.4025794 | 0.1607423 | 0.32148459 | All Treatment vs Str+pH | Day45 |
| Streptomyces | 3112.17965 | 0.39529119 | 0.45940318 | 0.86044504 | 0.38954377 | 0.51939169 | All Treatment vs Str+pH | Day45 |
| Agrobacterium | 11741.8041 | 0.74805667 | 0.57210176 | 1.30755877 | 0.19102301 | 0.52576544 | All Treatment vs Str+SynCom | Day45 |
| Pseudomonas | 3453.43072 | -0.2481343 | 0.804476 | -0.3084422 | 0.7577459 | 0.7577459 | All Treatment vs Str+SynCom | Day45 |
| Rhizobium | 2128.74471 | -0.5190221 | 0.67322664 | -0.7709471 | 0.44073828 | 0.58765104 | All Treatment vs Str+SynCom | Day45 |
| Streptomyces | 3112.17965 | 0.51437503 | 0.45942602 | 1.11960361 | 0.26288272 | 0.52576544 | All Treatment vs Str+SynCom | Day45 |
| Agrobacterium | 11741.8041 | 0.08403578 | 0.57208899 | 0.14689284 | 0.8832166 | 0.88979413 | All Treatment vs SynCom | Day45 |
| Pseudomonas | 3453.43072 | 0.47723744 | 0.80451529 | 0.59319871 | 0.55304817 | 0.88979413 | All Treatment vs SynCom | Day45 |
| Rhizobium | 2128.74471 | 0.18035085 | 0.67337769 | 0.26783016 | 0.78883006 | 0.88979413 | All Treatment vs SynCom | Day45 |
| Streptomyces | 3112.17965 | -0.0636549 | 0.45938745 | -0.1385647 | 0.88979413 | 0.88979413 | All Treatment vs SynCom | Day45 |
| Agrobacterium | 11741.8041 | -0.3610252 | 0.57205401 | -0.6311033 | 0.527973 | 0.703964 | Str vs Str+pH | Day45 |
| Pseudomonas | 3453.43072 | -0.1479177 | 0.80474725 | -0.1838064 | 0.85416536 | 0.85416536 | Str vs Str+pH | Day45 |
| Rhizobium | 2128.74471 | 0.77534668 | 0.67267011 | 1.15264031 | 0.24905805 | 0.65316149 | Str vs Str+pH | Day45 |
| Streptomyces | 3112.17965 | 0.450568 | 0.45928321 | 0.98102433 | 0.32658074 | 0.65316149 | Str vs Str+pH | Day45 |
| Agrobacterium | 11741.8041 | 0.64274717 | 0.57208065 | 1.12352546 | 0.2612144 | 0.2612144 | Str vs Str+SynCom | Day45 |
| Pseudomonas | 3453.43072 | -1.9686372 | 0.80465044 | -2.4465745 | 0.0144221 | 0.0576884 | Str vs Str+SynCom | Day45 |
| Rhizobium | 2128.74471 | 1.20051212 | 0.67271737 | 1.78457131 | 0.07433088 | 0.14866176 | Str vs Str+SynCom | Day45 |
| Streptomyces | 3112.17965 | 0.56965185 | 0.45930606 | 1.24024457 | 0.21488495 | 0.2612144 | Str vs Str+SynCom | Day45 |
| Agrobacterium | 11741.8041 | -0.0212737 | 0.57206788 | -0.0371874 | 0.97033558 | 0.9854456 | Str vs SynCom | Day45 |
| Pseudomonas | 3453.43072 | -1.2432655 | 0.80468972 | -1.5450247 | 0.1223403 | 0.2446806 | Str vs SynCom | Day45 |
| Rhizobium | 2128.74471 | 1.8998851 | 0.67286854 | 2.82356061 | 0.00474935 | 0.01899738 | Str vs SynCom | Day45 |
| Streptomyces | 3112.17965 | -0.0083781 | 0.45926748 | -0.0182422 | 0.9854456 | 0.9854456 | Str vs SynCom | Day45 |
| Agrobacterium | 11741.8041 | 1.00377233 | 0.57204199 | 1.7547179 | 0.07930757 | 0.15861515 | Str+pH vs Str+SynCom | Day45 |
| Pseudomonas | 3453.43072 | -1.8207195 | 0.80447945 | -2.2632269 | 0.0236217 | 0.09448682 | Str+pH vs Str+SynCom | Day45 |
| Rhizobium | 2128.74471 | 0.42516544 | 0.67269623 | 0.63203185 | 0.52736607 | 0.70315475 | Str+pH vs Str+SynCom | Day45 |
| Streptomyces | 3112.17965 | 0.11908384 | 0.45922672 | 0.25931384 | 0.7953931 | 0.7953931 | Str+pH vs Str+SynCom | Day45 |
| Agrobacterium | 11741.8041 | 0.33975143 | 0.57202923 | 0.59394069 | 0.55255178 | 0.55255178 | Str+pH vs SynCom | Day45 |
| Pseudomonas | 3453.43072 | -1.0953478 | 0.80451874 | -1.3614944 | 0.17335749 | 0.34671498 | Str+pH vs SynCom | Day45 |
| Rhizobium | 2128.74471 | 1.12453842 | 0.6728474 | 1.67131273 | 0.09465992 | 0.34671498 | Str+pH vs SynCom | Day45 |
| Streptomyces | 3112.17965 | -0.4589461 | 0.45918814 | -0.9994728 | 0.31756569 | 0.42342092 | Str+pH vs SynCom | Day45 |
| Agrobacterium | 11741.8041 | -0.6640209 | 0.57205587 | -1.1607623 | 0.24573857 | 0.36720005 | Str+SynCom vs SynCom | Day45 |
| Pseudomonas | 3453.43072 | 0.72537176 | 0.8044219 | 0.90173049 | 0.36720005 | 0.36720005 | Str+SynCom vs SynCom | Day45 |
| Rhizobium | 2128.74471 | 0.69937298 | 0.67289465 | 1.03934989 | 0.29864204 | 0.36720005 | Str+SynCom vs SynCom | Day45 |
| Streptomyces | 3112.17965 | -0.5780299 | 0.45921099 | -1.2587458 | 0.20812216 | 0.36720005 | Str+SynCom vs SynCom | Day45 |
